# Post-Plasma SiO_*x*_ Coatings of Metal and Metal Oxide Nanoparticles for Enhanced Thermal Stability and Tunable Photoactivity Applications

**DOI:** 10.3390/nano6050091

**Published:** 2016-05-13

**Authors:** Patrick Post, Nicolas Jidenko, Alfred P. Weber, Jean-Pascal Borra

**Affiliations:** 1 Institute of Particle Technology, Clausthal University of Technology, Leibnizstrasse 19, 38678 Clausthal-Zellerfeld, Germany; patrick.post@tu-clausthal.de; 2 Lab of Phys Gaz and Plasmas, CNRS, Univ. Paris Sud, CentraleSupelec, Université Paris-Saclay, F-91405 Orsay, France; nicolas.jidenko@u-psud.fr (N.J.); jean-pascal.borra@u-psud.fr (J.-P.B.)

**Keywords:** particle coating, silicon oxide, dielectric barrier discharge, gas phase, continuous process, TEOS, CVD

## Abstract

The plasma-based aerosol process developed for the direct coating of particles in gases with silicon oxide in a continuous chemical vapor deposition (CVD) process is presented. It is shown that non-thermal plasma filaments induced in a dielectric barrier discharge (DBD) at atmospheric pressure trigger post-DBD gas phase reactions. DBD operating conditions are first scanned to produce ozone and dinitrogen pentoxide. In the selected conditions, these plasma species react with gaseous tetraethyl orthosilicate (TEOS) precursor downstream of the DBD. The gaseous intermediates then condense on the surface of nanoparticles and self-reactions lead to homogeneous solid SiO_*x*_ coatings, with thickness from nanometer to micrometer. This confirms the interest of post-DBD injection of the organo-silicon precursor to achieve stable production of actives species with subsequent controlled thickness of SiO_*x*_ coatings. SiO_*x*_ coatings of spherical and agglomerated metal and metal oxide nanoparticles (Pt, CuO, TiO_2_) are achieved. In the selected DBD operating conditions, the thickness of homogeneous nanometer sized coatings of spherical nanoparticles depends on the reaction duration and on the precursor concentration. For agglomerates, operating conditions can be tuned to cover preferentially the interparticle contact zones between primary particles, shifting the sintering of platinum agglomerates to much higher temperatures than the usual sintering temperature. Potential applications for enhanced thermal stability and tunable photoactivity of coated agglomerates are presented.

## 1. Introduction

The coating of nanoparticles is an essential step to improve the particle thermostability [1], to create a protective layer between particle and environment [2] or to enhance particle dispersion in liquids. Silicon oxide is one of the preferred nontoxic and almost inert coating materials.

Many coating techniques use liquid phase reactions with additional steps such as washing, drying and separation [3]. Moreover, impurities from solvents remain in the final solid coatings. Therefore, gas phase coating techniques are generally preferred [4] (e.g., for atomic layer deposition [5], for flames [6,7] and for plasma enhanced chemical vapor deposition (CVD) [8]).

Most publications on SiO_*x*_ coatings deal with the coating of flat surfaces, but few groups used it to coat nanoparticles directly in the gas phase. Homogeneous coatings can be performed in non-continuous devices such as fluidized bed reactors [9]. In flame synthesis, direct coating of gas born nanoparticles such as TiO_2_, may be performed in a continuous one-step process.

A more versatile coating method is the CVD that can be induced by photochemistry [10] and plasmas. In dielectric barrier discharges (DBD), plasma filaments produce reactive species (electrons, photons, radicals, excited species and more stable ozone and nitrogen oxides in air), triggering the polymerization of organic and organo-metal precursors. For the coating of substrates, the precursors can be injected in the DBD or in post-DBD for SiO_*x*_ coatings [8,11], as well as for functional polymer coatings [12,13]. For the coating of supported particles by plasma, Mori *et al.* [14], Kogoma *et al.* [15] and Brüser *et al.* [16] used batch processes.

The direct SiO_*x*_ coating of suspended nanoparticles in gases using atmospheric pressure DBD is an emerging field [13]. Nessim *et al.* used HMDSO (hexamethyldisiloxane) and TEOS for the coating of particles either directly in a plasma as Vons *et al.* [17], or in post-DBD [18]. As for flat surface coating, the precursor injection in the DBD leads to a loss of functionality of the gaseous precursor reacting with plasma species in the gap, to particles electro-collection on surfaces in the gap and to discharge destabilization that hampers the economics of the process.

Here, post-DBD injection of the organo-silicon precursor is tested to avoid electrode coating so as to achieve stable DBD production of active species with subsequent controlled thickness of SiO_*x*_ coatings of nanoparticles *versus* tetraethyl orthosilicate (TEOS) concentration and reaction duration. The setup and operating conditions are presented. Preliminary tests confirm that plasma species trigger the post-DBD conversion of TEOS into solid SiO_*x*_ coatings of nanoparticles. DBD operating conditions are then scanned to produce ozone and dinitrogen pentoxide. In the selected conditions, subsequent sections present the coating of spherical particles and of agglomerates separately *versus* reaction duration and concentrations of reactants (plasma species and TEOS). Finally, properties of coated agglomerates for thermal stabilization of catalyst particles and for photoactivity control are depicted.

## 2. Experimental Section

**Figure 1:**
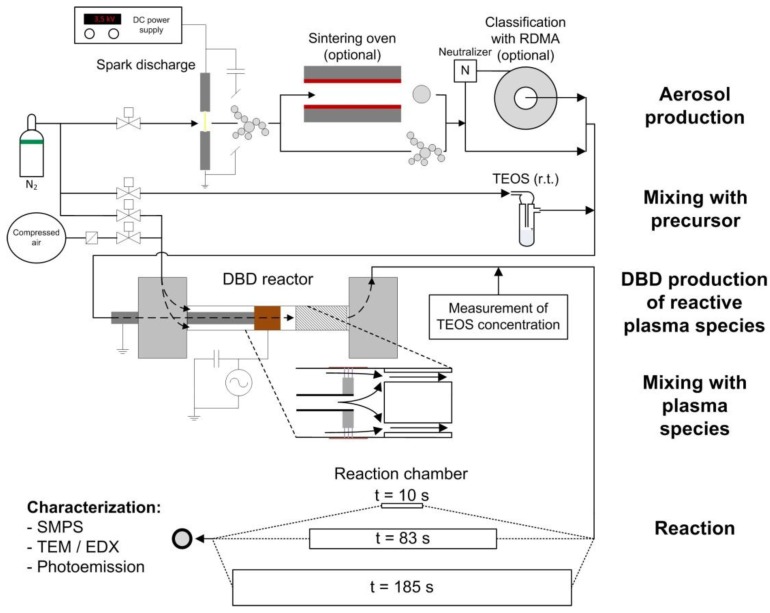
Experimental setup with the different process steps.

The experimental setup is shown in Figure 1. The first step is the production of nanoparticles with defined size, concentration and morphology (spheres or agglomerates). Then, a controlled amount of precursor is added. The third step is the post-DBD injection of this TEOS/nanoparticles aerosol, mixed with plasma species. Finally, post-DBD reaction times are tuned in different volumes.

### 2.1. Aerosol Production and Mixing with Precursor

**Figure 2:**
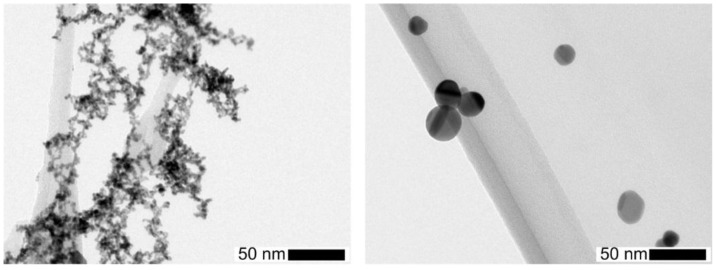
Effect of sintering on the morphology of platinum nanoparticles from the spark generator: (**left**) agglomerates downstream of the spark before sintering; and (**right**) spherical particles after sintering.

The metal core particles were produced in a spark discharge generator (SDG) without any side products in the gas stream [19]. Two electrodes of the same material with a 5 mm gap were polarized with a high voltage DC power supply. Then, repetitive sparks develop between the electrodes leading to vaporization and nucleation into nanoparticles [19]. The preferred material was platinum for its high TEM contrast and applications, e.g., in catalysis. Due to the high number concentrations of small primary particles large fractal agglomerates formed rapidly behind the SDG in 1 L/min N_2_ (standard liter per minute, nitrogen 5.0). Figure 2 (left) shows such agglomerates, converted into spherical particles (right) by sintering in a tube furnace at 1000 °C. Pt, Cu, Ti, Ni and Fe have been used in the spark generator to produce similar nanoparticles, agglomerated or spherical after sintering, which could be size selected with a Radial Differential Mobility Analyzer (RDMA) downstream of an X-ray source to establish a known charge distribution.

Among the organo-silicon precursors, tetraethyl orthosilicate (TEOS, Si(OC_2_H_5_)_4_) is one of the most prominent. Due to its non-hazardous character, TEOS is easy to handle. Moreover, its relatively low vapor pressure facilitates the addition of small amounts of gaseous precursor and it reacts more slowly compared to other precursors and is therefore suitable to achieve nano-sized SiO_*x*_ coatings.

The mixing of the TEOS with the particles is done in a simple T-piece prior to post-DBD injection. A second flow of nitrogen below 30 mL/min is used to transport the precursor vapor from a bottle. The nitrogen does not bubble through the precursor but flows over the surface of the liquid to prevent the formation and transport of droplets. In the bottle kept at room temperature (24 °C), the distance between the gas inlet and the surface of the initially liquid TEOS is kept constant at 5 mm.

The precursor concentration was measured by Fourier transform infrared spectroscopy (FTIR, Tensor 27, Bruker Optik GmbH, Ettlingen, Germany) on the Si–O absorbance near 1100 cm^−1^, directly after the mixing of all gas flows downstream of the DBD reactor (Figure 1), *i.e.*, at the entrance of the post-DBD reaction chamber. It can be tuned from 0.3 to 4.1 ppmv (below 0.2% of TEOS saturation vapor pressure of 242 Pa at 24 °C), without affecting significantly the total flow of 3 L/min (plus a maximum of 30 mL/min of the N_2_-TEOS mixture).

### 2.2. DBD Arrangement and Operating Conditions

The DBD is made of two stainless steel parts that hold a quartz glass tube with an inner diameter of 13 mm and a thickness of 1.5 mm serving as dielectric (see inset of Figure 1). On the outside of the glass tube, an electrode made of copper adhesive tape is connected to the high voltage AC power supply. A stainless steel disc (diameter: 12 mm, length: 2 mm) is fixed on the grounded central stainless steel tube in front of the polarized external electrode. Plasma filaments then occur in the 0.5 mm ring-shaped gap between the metal disc and the dielectric, in a so-called monoDBD [20].

To limit the electrode temperature below 100 °C, the generator frequency was fixed to 41 kHz. The voltage was measured with a HV probe. The current pulses related to each plasma filament were recorded via a 50 Ω input resistor to evaluate the charge and the energy per filament as well as the power, calculated, with *n* the number of periods and *T* the period duration, according to:

(1) $$P=\frac{1}{n\cdot T}\cdot \int_{nT}u\left(t\right)\cdot i\left(t\right)dt$$

### 2.3. Mixing with Plasma Species and Reaction

The particles and the precursor flow through a stainless steel tube (4.57 mm inner diameter) without contact with the plasma. The combined aerosol flow from the spark generator and the TEOS bottle was injected into the inner tube at 1 L/min N_2_ varying only slightly with the small amount of precursor gas flow. A 1:1 mixture of nitrogen and filtered air was injected at 2 L/min into the DBD gap to transport plasma species downstream of the DBD up to the post-DBD mixing with the aerosol/precursor mixture. The total flow of about 3 L/min was injected in the post-DBD reaction volume. To enhance the mixing of plasma species and of the particle/TEOS aerosol flow, a static mixer was placed about 8 mm downstream of the DBD so that there is no reflux of TEOS into the discharge zone. As shown in Figure 1, it deflects the inner flow towards smaller tubes near the outer diameter of the mixer, leading to turbulent mixing of the two gas flows.

The influence of the reaction duration was tested with different tubes as reaction chambers with laminar flow leading to defined transit time distributions. Unless stated otherwise, the selected conditions defined from the preliminary results detailed in Section 3.2, are specified in Table 1.

### 2.4. Aerosol Characterization

Downstream of the reaction chamber, the coated particles were collected at 0.2 L/min in a bypass across a lacey transmission electron microscopy (TEM) grid for 2 min. As a consequence, the particles analyzed with the TEM refer to different ages since condensation and heterogeneous reactions still happen during the 2 min collection. However, for the presentation of the experimental data, the beginning of the sampling was chosen as time reference. Then, the TEM grid was removed and stored in ambient air for at least 20 min before being transferred under ultra-high vacuum into the TEM. Coupled with the energy-dispersive X-ray spectroscopy (EDX) sensor, size, coating thickness and atomic composition of the coated particles were characterized.

On-line measurements of the particle number size distributions were obtained with a scanning mobility particles sizer (SMPS Grimm 5.403, GRIMM Aerosol Technik GmbH & Co. KG, Ainring, Germany). Assuming spherical particles, the coating thickness is defined as half the difference of the mobility equivalent mode diameters of non-coated and coated particles.

The coated surface of particles was estimated by aerosol photoemission measurements (APE) [21]. Downstream of an electrostatic precipitator, the neutral aerosol exposed to UV is charged positively due to electron emission from metal nanoparticles. The aerosol charge, measured with a Faraday Cup Electrometer after an ion trap is proportional to the uncoated active surface area.

## 3. Results and Discussion

### 3.1. DBD Electrical Characterisation and Post-DBD Ozone and NO_x_

**Figure 3:**
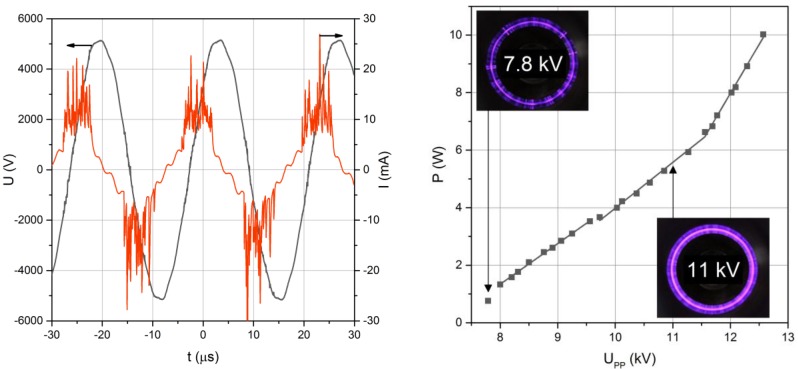
Temporal evolution of voltage and dielectric barrier discharge (DBD) current (**left**) and DBD power *versus* peak-to-peak voltage characteristic of the DBD with insets of violet light emitted from plasmas in air (**right**).

As shown in Figure 3 (left), both voltage and current are sinusoidal with a phase shift between them. The real discharge current with pulses related to each plasma filament is superposed to this sinusoidal capacitive current. From the instantaneous voltage and current, the discharge power was calculated as detailed above and depicted in Figure 3 (right) *vs.* applied voltage.

At increasing voltages, the plasma filaments originate from the edges of the inner electrode until they cover the whole surface of the smaller electrode for higher applied voltages (see insets of Figure 3). As expected for such asymmetrical mono-DBD [20], negative current pulses for filaments developing from the metal electrode to the dielectric surface are slightly larger than positive pulses related to the same streamer-like plasma filament, then directed towards the central metal electrode.

Additional post-DBD FTIR analysis of gaseous products without any TEOS reveal ozone and NO_*x*_, respectively, from 0 to 301 ppmv for ozone and from 35 to 114 ppmv for NO_2_, N_2_O, N_2_O_5_ and HNO_*x*_, without NO, as depicted in Table 2 for different voltages. The species were quantified from the adsorption signal at the corresponding wavelengths of each species: O_3_: 1050 cm^−1^, NO_2_: 1630 cm^−1^, N_2_O: 2340 cm^−1^, N_2_O_5_: 1240 cm^−1^, NO: 1890 cm^−1^ and HNO_*x*_: 1330 cm^−1^. These species were expected from a DBD fed with a 10% O_2_/90% N_2_ mixture, since these DBD were used as ozonizers.

Finally, it can be noted from electrode temperatures depicted in Table 2 that up to 10 kV the gas is not heated above 100 °C in the gap of the DBD since ozone destruction would be faster than its production. On the contrary, at 12 kV, ozone and dinitrogen pentoxides are converted into other NO_*x*_, as expected for higher gas temperatures arising from higher electrode temperatures.

In the selected arrangement, a stable DBD with constant energy and number of pulses per cycle leads to constant related post-DBD concentrations of plasma species at the entrance of the reaction volume. These concentrations can be tuned with DBD operating conditions with ozone and N_2_O_5_ up to 10 kV or only with other nitrogen oxides at 12 kV, for reaction temperatures near ambient temperatures up to 35 °C for 8 and 10 kV and below 70 °C at 12 kV, respectively.

### 3.2. Proof of Concept and Ranges of TEOS Concentration and Reaction Duration for Nanoparticle Coating

It is first confirmed that post-DBD gas phase reactions of ozone and dinitrogen pentoxide plasma products with gaseous TEOS precursor lead to final homogeneous SiO_*x*_ coatings.

**Figure 4:**
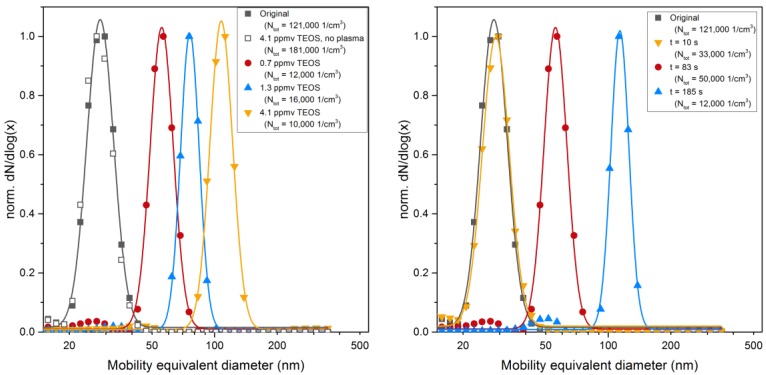
Mobility equivalent diameter measured with scanning mobility particles sizer (SMPS): (**left**) as a function of the tetraethyl orthosilicate (TEOS) concentration at constant reaction time *t*_Reaction_ = 83 s; and (**right**) as a function of reaction time at constant TEOS concentration of 0.7 ppmv (the total number concentration *N*_tot_ is given in the legend).

This can be stated from EDX analysis presented below in Table 3 and from Figure 4 (left). The size distributions of sintered particles do not evolve by mixing with TEOS precursor unless plasma filaments develop in the DBD. Then, a significant increase in diameter is found when the DBD is fed with a 10% O_2_/90% N_2_ mixture (Figure 4, left), while in pure nitrogen, *i.e.*, without oxygen injected either in the DBD or in post-DBD, some holey heterogeneous coatings are formed. As sub-second transit time from the DBD gap (where plasma filaments produce electrons, photons, radicals, excited species) to the entrance of the reaction volume is longer than the times of life of these plasma species (e.g., for atomic oxygen—10^−8^ s for O^1^d, 10^−8^ to 10^−7^ s for O^3^p- and metastable O_2_^1^∆g and vibrationally excited N_2_, up to 10^−3^ s, in air [22]), only stable species such as ozone and nitrogen oxides are analyzed in post-DBD at the entrance of the reaction volume and could react with TEOS.

Actually, Okuyama *et al.* [23] and Fujino *et al.* [24] studied the kinetics of TEOS and ozone reactions, which was later modeled by Romet *et al.* [25]. Atomic oxygen produced by ozone decomposition is suspected to be the main starter of the reaction [25]. One critical step is the gas phase conversion of TEOS into triethoxysilanol (Si(OC_2_H_5_)_3_OH), then condensed on the surface and finally converted into solid SiO_*x*_. A simple model may describe the overall reaction between TEOS and ozone as [25]:

$$\text{Si}{\left({\text{OC}}_{2}H_{5}\right)}_{4}+4\;O_{3}\to {\text{SiO}}_{2}+4\;{\text{CH}}_{3}\text{CHO}+2\;H_{2}O+4\;O_{2}$$

**Figure 5:**
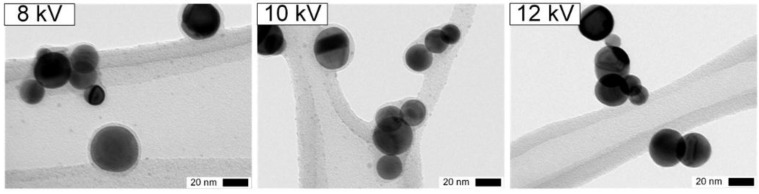
Transmission electron microscopy (TEM) micrograph of final solid coating of nanoparticles for different applied voltage (*U*_pp_): (**left**) 8 kV; (**middle**) 10 kV; and (**right**): 12 kV, for 0.4 ppmv TEOS and *t*_Reaction_ = 185 s.

The coatings formed at different voltages are shown in Figure 5. Solid SiO_*x*_ are formed up to 10 kV, contrary to 12 kV when ozone and N_2_O_5_ are not present anymore downstream of the DBD (Table 2 and Figure 5, right). Similar homogeneous 2 nm coatings at 8 and 10 kV probably result from the similar ozone concentrations for both voltages (Figure 5, left and center).

The importance of ozone is further corroborated by a decrease in its concentration when TEOS is added to the system. FTIR measurements analogous to those described above yield an ozone concentration of only 55 ppmv at 10 kV, which is significantly lower than without precursor (301 ppmv). On the other hand, the N_2_O_5_ concentration of 19 ppmv is comparable to that measured without TEOS (24 ppmv). The addition of Pt particles to the system shows no further change in the ozone concentration (55 ppmv), but N_2_O_5_ decreases somewhat to 15 ppmv. While these findings suggest that ozone is the most important species for the reaction with TEOS in this process, further research is required to understand all possible reaction pathways.

Finally, since atomic oxygen can still be produced from slow post-DBD reactions of ozone and N_2_O_5_, it is not yet clear if TEOS reacts directly with ozone or with atomic oxygen to form condensable intermediate species.

#### 3.2.1. Coating Composition

For two coating thicknesses controlled by the reaction time (Table 3), the particles were analyzed by EDX showing contributions of Pt (core particles) and Cu from the TEM grid and sample holder as well as C, O and Si. While Si and O obviously belonged to the SiO_*x*_ coatings, the source of C at least partly arises from the carbon coating of the TEM grid. As expected, the ratio of the coating elements to the core material increases for thicker coatings. The ratio of Si to O is nearly stoichiometric SiO_2_ with a slight excess of oxygen. To determine the actual carbon content, coated Pt particles were deposited on a Mo TEM grid without a C film. The experiment showed that there is indeed some amount of carbon in the coated particles. The chemical composition of the coating following from this measurement is SiO_2.3_C_1.5_H_z_. The H content cannot be measured with EDX.

#### 3.2.2. Ranges of TEOS Concentration and Reaction Duration for Nanoparticle Coating

With the selected DBD arrangement polarized with applied voltages up to 10 kV at 3 L/min, different TEOS concentrations and reaction times have been tested. The goal was to define the ranges of conditions for the parametric study to control the thickness of nanometer sized coating, detailed in the next sections.

**Figure 6:**
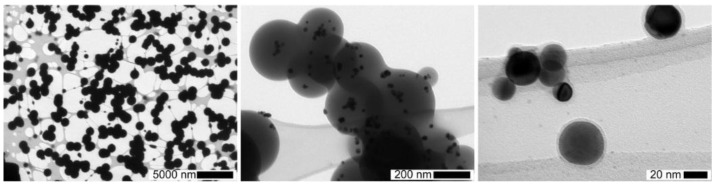
TEM micrographs: (**left**) Large SiO_*x*_ particles (1.13 µm) formed in the gas phase during the reaction duration in the 20 L tank (*U*_pp_ = 10 kV, with post-DBD injection of *c*_TEOS_ = 1400 ppmv without Pt nanoparticles); (**middle**) smaller sub-micron sized SiO_*x*_ coated Pt particles, formed and agglomerated in the gas phase (*U*_pp_ = 8.6 kV, 20 L tank with 280 ppmv TEOS and Pt nanoparticles); and (**right**) nanometer thick coatings of Pt nanoparticles (*U*_pp_ = 8 kV, *t*_Reaction_ = 185 s with 0.4 ppmv TEOS).

For longer reaction durations, a 20 L tank was used with a broad residence time distribution, varying from a few minutes up to several tens of minutes. With this tank downstream of the DBD and the highest TEOS concentrations (1400 ppmv), high concentrations of micron-sized spherical dense SiO_*x*_ particles were collected on TEM grids even without Pt nanoparticles from the spark generator (see Figure 6, left.). In that case, post-DBD heterogeneous nucleation of condensable gaseous intermediates probably occurs on single digit nano-sized particles and/or on ions reported downstream of DBDs [22,26,27]. Lowering the TEOS concentrations and reaction time lead to much smaller sub-micron-sized SiO_*x*_ coating (Figure 6, center), down to nanometer thick homogeneous coatings on 30 nm Pt nanoparticles (Figure 6, right).

For different reaction durations and TEOS concentrations, TEM and EDX analyses confirm that SiO_*x*_ particles are always formed in the gas phase, even if slow reactions of SiO_*x*_ formation still occur after collection near ambient temperature, as proved in the next section. Such spherical SiO_*x*_ particles usually arise from liquid droplets [4,7].

As the precursor concentration is below saturation (<0.2% of *p*_sat TEOS_), these droplets cannot be formed by TEOS condensation. Indeed, the size distributions measured on line downstream of the reactor with SMPS, are the same for spherical sintered Pt nanoparticle with or without TEOS (see Figure 4, left). These droplets are thus formed by condensation of gaseous intermediate species continuously formed by TEOS reactions in post-DBD, up to the onset of condensation.

Hence, nanoparticles can be coated with SiO_*x*_ by a three step process starting with gas phase reactions of TEOS with post-DBD ozone and dinitrogen pentoxides, producing intermediate species more condensable than the TEOS precursor. In a second step, droplets are formed by condensation of silanol-like intermediates on nanoparticles, then converted by sol-gel reactions into solid SiO_*x*_ coatings. Finally, coagulation discussed below sometimes leads to agglomerated coated particles.

As expected from kinetic considerations, the growth rate of coating and the related thickness depend on the reaction conditions, including temperature and reactants concentrations downstream of the DBD (ozone and N_2_O_5_ plasma species, as well as TEOS). From a practical point of view, for fixed DBD operating conditions, the reaction time and the precursor concentration control the SiO_*x*_ coating thickness. To avoid the coating of the TEM grid, low TEOS concentrations in the ppmv range will be used. In this way, nanometer coating thickness can be achieved, as detailed in the next sections.

### 3.3. Homogeneous Nanometer-Sized Coating of Spherical Nanoparticles

To investigate the influence of particle properties and operational parameters on the coating thickness, size-selected sintered spherical Pt nanoparticles were used. TEOS concentrations and the reaction time were varied and the coating thickness was determined for different materials.

As outlined above, during the reaction of TEOS vapor with the plasma species intermediate molecules are formed which condense on the particle surfaces. Since the Knudsen number (*Kn*), defined as twice the mean free path (λ ≈ 65 nm) of the gas molecules divided by the particle diameter, is significantly larger than 1 (*Kn* ≈ 4), the condensation takes place close to the free molecular regime (*Kn* >> 1). In this case the theory for heterogeneous condensation [28] predicts that the particle diameter increases linearly with the concentration of the condensing vapor and with the residence time as long as the concentration of the condensing vapor is constant, *i.e.*, without depletion:

(2) $$\Delta R=\frac{M\cdot (p-p_{S})}{\rho_{l}\cdot N_{A}\cdot \sqrt{2\pi mkT}}\cdot \Delta t$$

where ∆*R* = coating thickness, *M* = molecular weight of the liquid, *p* = partial pressure of condensing vapor, *p*_S_ = partial pressure at particle surface, ρ_l_ = liquid density, *N_A_* = Avogadro number, *m* = mass of vapor molecule, and *kT* = thermal energy.

#### 3.3.1. Kinetics of TEOS Conversion into SiO_*x*_ Coating

Although the mechanisms and the kinetics of the formation reactions leading to the condensing species are not known in detail, our results support that such depletion of intermediate condensable species does not affect condensation here, even though it necessarily happens along the reaction volume, while post-DBD plasma species are consumed by reaction with TEOS. However, without a final forced condensation step by cooling, all formed intermediates do not condense. The results shown in Figure 4 and in Figure 8 confirm that free molecular condensation is a reasonable approximation to describe the thickness of the liquid coating *versus* TEOS concentration and reaction time, as detailed below.

The size distribution of particles/drops suspended in carrier gas was first measured with the SMPS at ambient conditions before collection, *i.e.*, for the reference time of reaction, while TEM analyses were performed on particles with residual solid coating only, formed after additional time delay of 2 min maximum for particles collected immediately and at least 20 min in air before final forced evaporation under vacuum conditions for TEM analysis. Therefore, the TEM results show the layers having a vapor pressure low enough to withstand the vacuum, *i.e.*, solid coatings.

**Figure 7:**
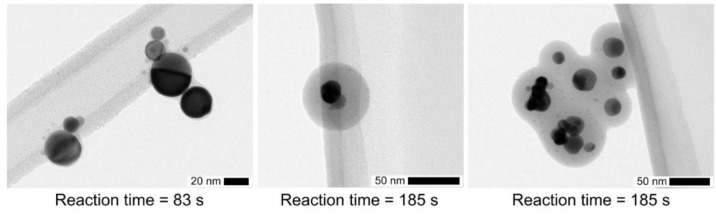
TEM micrographs showing the time dependence of the coating thickness for a constant TEOS concentration of 0.7 ppmv.

**Figure 8:**
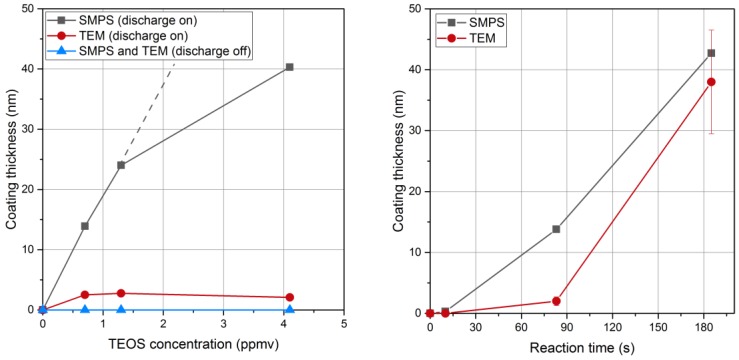
Coating thickness measured with scanning mobility particles sizer (SMPS) and TEM as a function of TEOS concentration for *t*_Reaction_ = 83 s (**left**) and as a function of the reaction time for 0.7 ppmv TEOS concentration (**right**).

Some examples of TEM micrographs are shown in Figure 7 for a TEOS concentration of 0.7 ppmv and different reaction times. While the solid coating for a reaction time of 83 s is only 2.5 nm thick, it increases to 38 nm after 185 s (Figure 8, right). For longer reaction times, agglomeration may take place as shown in Figure 7 (right). Agglomeration may occur by diffusion or by electrical forces between oppositely charged particles, since bipolar ions are produced downstream of such a DBD, which can even be used as an aerosol neutralizer [26,27].

Particles and a few particle-clusters are well separated with smeared boundaries so that the coating thickness may only be deduced from the outside contour. This supports that core particles are not mobile in the coating, *i.e.*, the core is surrounded by a solid or at least more viscous gel shell. However, at the moment of collision the surface is liquid-like leading to conforming contacts. This is confirmed from the larger coating of drops/particles (measured before collection with SMPS) in comparison with the residual solid coating, formed after additional time delay before the final forced evaporation under vacuum conditions for TEM analysis (Figure 8), detailed below.

For a reaction time of 83 s, the coating thickness measured with SMPS increases with the TEOS concentration in qualitative agreement with Equation (1) (Figure 8, left). The deviation from the linear relationship at higher TEOS concentration may indicate that the concentration of the condensing vapor species is not directly proportional to the TEOS concentration. The reaction time of 83 s seems too short to complete the conversion of all the liquid coating into a solid silica coating. The reaction of formation of solid SiO_*x*_ is thus not limited by the amount of intermediate (unless much smaller precursor concentrations are used) but rather by a too short reaction time. Then, larger coatings could only be achieved for longer reaction times, as depicted below.

For longer reaction times, the thickness of the solid coating approaches the one of the liquid coating measured with SMPS (Figure 8, right). For a TEOS concentration of 0.7 ppmv the thickness of the liquid coating measured before collection increases with time corroborating Equation (1). An induction period is reported here before condensation linearly increases with time, as for the condensation of organic vapors on metal nanoparticles [29]. The solid coating also increases with time. However, reaction times of a few minutes are required to convert all the liquid intermediates into the final solid coating with the closest approach of thicknesses measured with SMPS and TEM, respectively. Conversion of liquid intermediates into solid SiO_*x*_ coatings might still occur after collection as already reported [25]. Actually, much slower rates of conversion are expected here with post-DBD reaction temperatures close to ambient temperature, than in classical sol-gel process for the formation of oxides usually performed above a few hundred degrees Celsius [4].

Hence, the first gas phase reaction and the second condensation step leading to suspended droplets of intermediates are faster than the final self-reaction of silanol-like intermediates leading to the solid silica coating, for the near ambient temperature of the reaction. The step limiting the overall reaction rate is the conversion of the condensed intermediates into solid silica coating. The thickness of final solid SiO_*x*_ coatings can be controlled from a nanometer to few tens of nanometers by playing on TEOS concentration for reaction times longer than 3 min, so that the SiO_*x*_ formation is nearly completed before collection, and on reaction times for TEOS concentrations in the ppmv range.

#### 3.3.2. Influence of the Particle Material on the Coating Thickness

**Figure 9:**
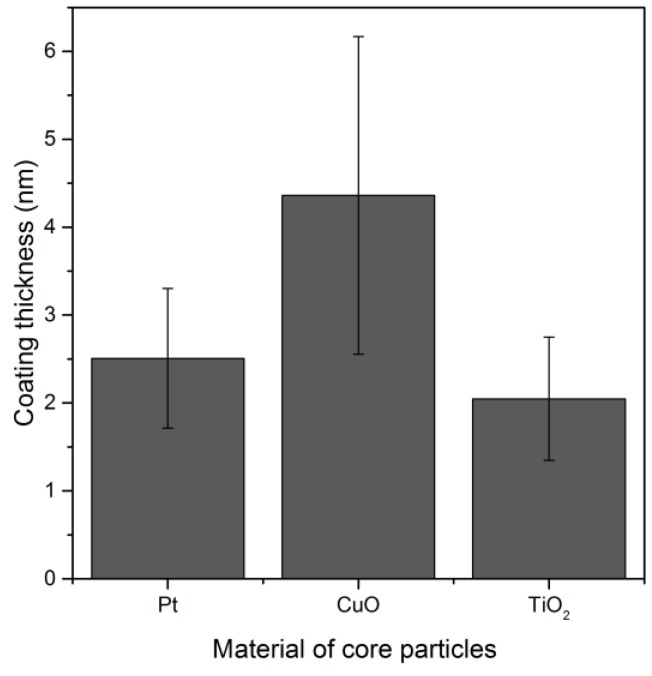
Influence of the particle material on the coating thickness from TEM analysis at a given TEOS concentration of 0.7 ppmv for *t*_Reaction_ = 83 s.

Different electrode materials (Pt, Cu, Ti, Ni, Fe) were used in the SDG producing different metal nanoparticles, sintered and coated without prior size classification. Contrary to the inert Pt, other metal nanoparticles were oxidized. A homogeneous SiO_*x*_ coating was created on spherical platinum, copper oxide and titanium oxide particles (Figure 9).

For the nickel and iron oxide particles, complete coalescence to spherical particles was not achieved so that the coating was performed with agglomerates. A very thin SiO_*x*_ coating was found on the outer surface of these particles, while most of the SiO_*x*_ was deposited inside the agglomerates. Therefore, the thicknesses of such heterogeneous coatings on nickel and iron oxide agglomerates have not been plotted here. The coating of agglomerates will be discussed in the next section.

#### 3.3.3. Influence of the Particle Concentration on the Coating Thickness

For given TEOS concentration assumed to be related to the gas phase concentration of intermediates (*N*_vap_), the coating thickness could also be controlled *versus* the total concentration of particles (*N*_part._) affecting the amount of condensable species per particle (*N*_vap_/*N*_part._). The depletion effect could only be addressed for longer reaction times (>3 min) and smaller TEOS concentrations with a final forced condensation. Similar coating thickness was found even though the particle number concentrations differed by about two orders of magnitude for classified and non-classified Pt particles and for the different materials tested.

### 3.4. Heterogenous Coatings of Agglomerates

While spherical nanoparticles facilitate the investigation of the coating kinetics (“idealized system”), nanoparticles in technical applications are mostly encountered as agglomerates consisting of much smaller primary particles as shown in Figure 2 (left). The concave surface areas in the necks between individual primary particles as well as in the interior of the highly ramified agglomerates are most prone for condensational effects inducing capillary condensation [29]. Therefore, the coating of nanoparticle agglomerates is a superposition of structure-induced deposition and reaction to form SiO_*x*_ layers. Since deposition and coating inside the agglomerates will affect much less the agglomerate mobility compared to the coating of a sphere, the SMPS measurements can only provide a qualitative picture of the coating progress. Thus, another surface sensitive *in situ* technique is used here to follow the degree of surface coverage, *i.e.*, the aerosol photoemission (APE) [29]. Since SiO_2_ has a high photothreshold (>10 eV [30]) that can only be overcome by extreme short wavelength UV light (far below UV-C), silica coatings are commonly employed to inhibit photocatalytic effects of sun blocking nanoparticles such as ZnO or TiO_2_ [1] by suppressing the exchange of photogenerated species with the environment [2].

**Figure 10:**
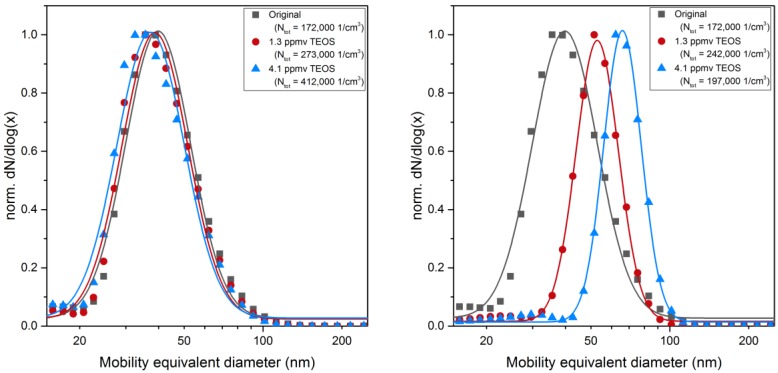
Change in mobility equivalent diameter depending on the precursor concentration without (**left**) and with (**right**) plasma for agglomerates *t*_Reaction_ = 83 s).

As starting point for the coating of Pt nanoparticle agglomerates the condensation of pure TEOS vapor without plasma (Figure 10, left) and with plasma turned on (Figure 10, right) was investigated as a function of the precursor concentration by tandem DMA measurements.

As shown in Figure 10 (left), small variations of the agglomerate size distribution were observed without a pronounced shift when the plasma was turned off. However, while with the plasma turned on a systematic shift of the size similar to the behavior of spherical particles was observed, the equivalent layer thickness appears to be smaller in comparison. On the one hand, the available surface area for the agglomerates is substantially higher (about a factor of 3) compared to the spherical Pt particles due to surface losses of the Pt particles as a consequence of coalescence but also due to particle losses in the sintering furnace. Therefore, the amount of condensing species per particle surface favors thicker layers on spherical particles. On the other hand, as outlined above, substantial condensation occurs inside agglomerates with a minor effect on the measured agglomerate mobility.

**Figure 11:**
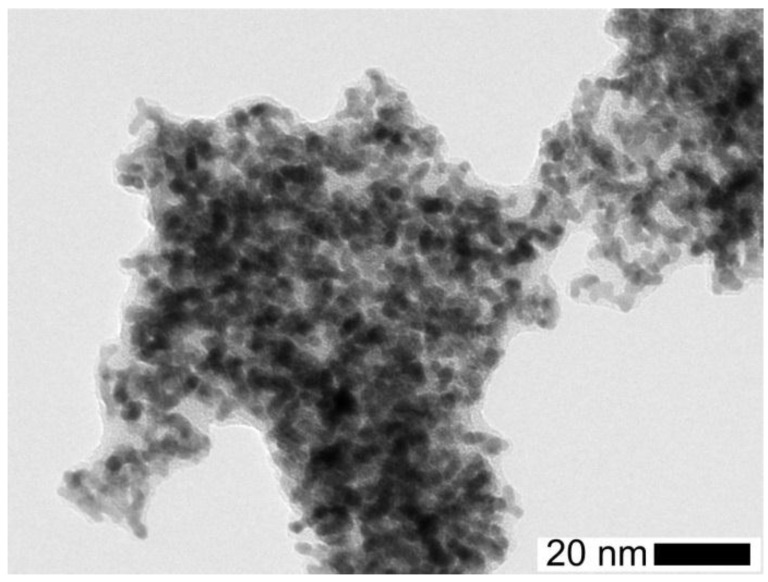
TEM micrograph of a coated Pt agglomerate after a reaction time of 185 s with a TEOS concentration of 0.7 ppmv showing a thick coating.

Next, the morphology of the solid silica coatings of the Pt agglomerates was studied with TEM for different TEOS concentrations and different reaction times. The coating in Figure 11 covers nearly the whole agglomerate. As already found for the spherical particles, the coating thickness of the agglomerates depends more on the reaction time than on the precursor concentration (*cf.* Figure 8).

**Figure 12:**
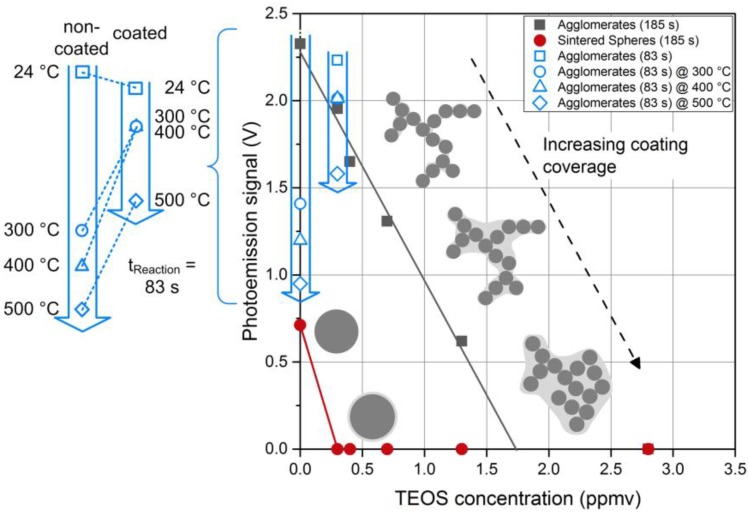
Measurement of the coating coverage by photoemission for Pt particles and sketches of the different kinds of observed coatings from TEM micrographs (*t*_Reaction_ = 185 s). Additionally, the open, blue symbols show the change in APE signal depending on the sintering temperature, analogous to the particles shown in Figure 13 (*t*_Reaction_ = 83 s).

In order to follow the coating kinetics, photoemission measurements were done for Pt agglomerates as well as spheres as a function of the precursor concentration (Figure 12). The non-coated agglomerates (*c*_TEOS_ = 0) gave the highest signal corresponding to the largest amount of active surface area. Uncoated sintered spheres gave a smaller signal due to a reduced particle surface area, a consequence of particle losses (number concentration) and surface loss due to sintering. For the spheres, a small amount of TEOS is sufficient to obtain a complete coating, reflected in a rapid drop of the APE signal. Higher precursor concentrations increased the coating thickness, but even a small, hermetic coating was enough to prevent photoemission. Agglomerates, however, behaved differently in the way that coating was favored in the necks and the gaps between branches, leaving some outer particles exposed. The fraction of the uncoated primary particles contributing to the APE signal decreased with increasing TEOS concentration. At high TEOS concentrations the whole agglomerate was covered. Parallel to the complete coating of the agglomerates a restructuring of some branches to more compact structures was observed due to capillary forces. Such a compactization of nanoparticle agglomerates is well documented in the literature [31]. However, at lower precursor concentrations, restructuring did not occur. This observation raises the question if the mere coating of the necks would lead to mechanical stabilization against restructuring without hindering mass transport processes, which would enable interesting applications such as catalysis at higher temperatures.

Silica coatings have been employed in various forms to protect noble metal nanoparticle catalysts from sintering [32]. In this context, two opposing effects have to be managed: On the one hand, the thermal stability of the nanoparticles increases with increasing coating thickness. On the other hand, thicker silica layers hamper the mass transport across the coating reducing the catalytic activity. To overcome this problem, one solution is to obtain a sufficient porosity of the coating by high temperature treatment [1] or by adding pore building agents [32]. However, an easier and more versatile approach would be the precise control of the coating process by depositing silica only in the necks between the primary particles. Such a technique was applied by Kim and Ehrman [33] using TEOS as precursor to coat TiO_2_ nanoparticles.

**Figure 13:**
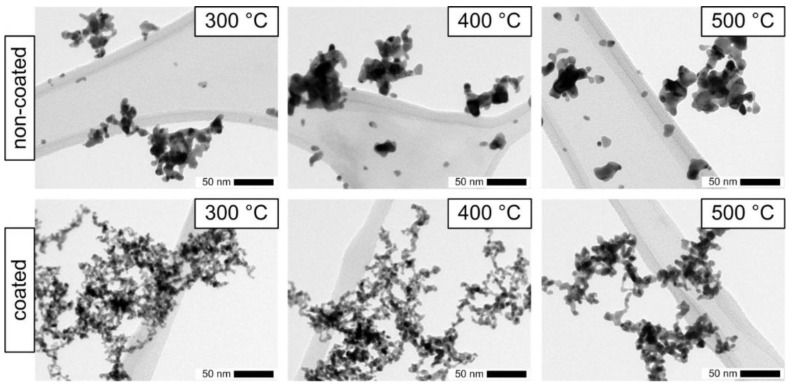
TEM micrographs showing the sintering behavior of non-coated (**top**) and coated (**bottom**) Pt particles during the transit through a tube furnace (residence time at 24 °C of 8 s).

To test if such a precise control of the coating can also be realized in the post-plasma process investigated here minute amounts of TEOS precursor (0.3 ppmv) and short reaction times (83 s) were used. As a measure for the surface coverage, the APE signal of the coated Pt agglomerates was determined and compared to the uncoated agglomerates. As indicated in Figure 12 (open, blue symbols), the photoemission activity was only reduced by about 5% due to the silica coating (silica confirmed by EDX). It was verified by TEM microscopy that at these conditions most of the silica was in fact deposited in the necks between the primary particles leaving most of the Pt surface accessible for interaction with the surrounding gas molecules. In order to test if this coating was sufficient to stabilize the agglomerates against thermal restructuring, the sintering behavior of non-coated and coated particles was studied by passing the aerosol particles through a tube furnace at a constant gas flow rate either directly after the SDG or after coating. Figure 13 shows the TEM micrographs for furnace temperatures of 300, 400 and 500 °C, respectively. The applied coating, which conserved the surface functionality as shown above, was very thin and non-homogeneous. With increasing temperature, the differences in the morphologies of the coated and non-coated agglomerates became more pronounced. While the primary particles of the non-coated agglomerates visibly sintered to larger diameters even at temperatures as low as 300 °C, the coated particles showed much smaller changes and retained a branched structure. The thin coating did not completely prevent the sintering of primary particles but it preserved a higher amount of surface area. As indicated by the blue symbols in Figure 12, also the photoactivity of the coated Pt agglomerates was only reduced by 12% for temperatures up to 400 °C and by 29% for a temperature of 500 °C. The uncoated agglomerates start with a slightly higher initial photoactivity at room temperature (24 °C), which decreases at elevated temperatures much faster compared to the coated particles (*cf.* insert in Figure 12). At 500 °C the remaining photoactivity of the coated particles is about 60% higher than the one of the uncoated Pt agglomerates. However, the coating process was not optimized so far and additional experiments will be necessary to explore the potential and to define the limits of this coating process for catalyst particles. Nevertheless, the first results of the post-plasma coating are promising and suggest further applications. Indeed, the operating conditions can be tuned to cover preferentially the interparticle contact zones between primary particles within agglomerates. This hampers the sintering of platinum agglomerates, retaining high apparent surface to much higher temperatures than usual sintering temperatures (e.g., for TiO_2_ agglomerates that could be heated more than 400 °C above the usual sintering temperature [1]).

## 4. Conclusions

Homogeneous SiO_*x*_ coatings of spherical and agglomerated metal and metal oxide nanoparticles (Pt, CuO, and TiO_2_) have been achieved in post-DBD. EDX results suggest some amount of carbon in the coating.

It has been shown that non-thermal plasma filaments induced in a dielectric barrier discharge (DBD) trigger post-DBD gas phase reactions of ozone and dinitrogen pentoxide with gaseous TEOS precursor, forming the final homogeneous SiO_*x*_ coatings.

This confirms the interest of post-DBD injection of the organo-silicon precursor to avoid electrode coating to achieve stable DBD production of active species with subsequent controlled thickness of SiO_*x*_ coatings of nanoparticles *versus* TEOS concentration and reaction time.

The coating thickness can be controlled through the reaction time and the precursor concentration. At this point, it has to be underlined that larger coating thicknesses were measured before collection in the gas phase from size distribution measurements than after collection by TEM analysis. A possible explanation is the formation of a liquid intermediate product, while the subsequent reaction to a solid SiO_*x*_ coating requires more time and could still happen after collection.

The process seems independent of the particle material, so that very homogeneous coatings can be achieved on nanoparticles from different materials and with different structures (single spherical particles as well as agglomerates). Even thermally unstable materials could be coated at near ambient temperatures.

For agglomerates, the operating conditions can be tuned to cover preferentially the interparticle contact zones between primary particles. This hampers the sintering of platinum agglomerates, retaining high apparent surface to much higher temperatures than usual sintering temperatures. The coatings are suitable for enhanced thermal stability, as well as for tunable photoactivity.

Post-plasma chemistry is complex because of numerous possible pathways, and more analysis in different plasma and mixing conditions would be required to identify all reaction pathways. Then, the growth rate of the coating and the related coating thickness could be defined *versus* conditions of reaction (temperature and concentrations of reactants—plasma species and TEOS—as well as the relative concentration of intermediate condensable species and nanoparticles—*N*_condensing vap_/*N*_part_), probably also controlling the amount of liquid condensed per particle, and thus the final solid coating thickness.

## Figures and Tables

**Table 1:** Summary of the standard experimental conditions.

Description	Abbr.	Value
Volume flow through discharge	*Q*_Plasma_	2 L/min
Volume flow in particle generation step	*Q*_Particles_	1 L/min
TEOS concentration	*c*_TEOS_	0.7 ppmv
Oxygen concentration in discharge	*c*_Oxygen_	10.5%
Applied peak-to-peak voltage	*U*_PP_	10 kV
Applied plasma power (Figure 3)	*P*_Plasma_	4 W
Reaction time before sampling	*t*_Reaction_	83 s

**Table 2:** Electrode and reaction temperatures, and ozone and NO_*x*_ concentrations (in ppmv) for three DBD voltages. * HNO_*x*_ could not be quantified due to significant interference.

*U*_DBD, PP_ (kV)	*T*_electrode_/*T*_reaction_ (°C)	O_3_	NO_2_	N_2_O	N_2_O_5_	NO	HNO_x_	Total NO_*x*_
8	33/20	267 ± 9	23 ± 8	2 ± 2	10 ± 0	0	*	35
10	46/35	301 ± 3	20 ± 0	20 ± 4	24 ± 0	0	*	64
12	120/<70	0	76 ± 5	38 ± 4	0	0	*	114

**Table 3:** Elemental composition from the quantitative energy-dispersive X-ray spectroscopy (EDX) analysis as a mean of several measurements on the samples for two different reaction times.

Element	*t*_Reaction_ = 83 s	*t*_Reaction_ = 185 s
Si	5 at.%	23 at.%
O	13 at.%	48 at.%
C	30 at.%	23 at.%
Pt (core)	53 at.%	6 at.%
